# Excitatory Pathways from the Lateral Habenula Enable Propofol-Induced Sedation

**DOI:** 10.1016/j.cub.2017.12.050

**Published:** 2018-02-19

**Authors:** Cigdem Gelegen, Giulia Miracca, Mingzi Z. Ran, Edward C. Harding, Zhiwen Ye, Xiao Yu, Kyoko Tossell, Catriona M. Houston, Raquel Yustos, Edwin D. Hawkins, Alexei L. Vyssotski, Hailong L. Dong, William Wisden, Nicholas P. Franks

**Affiliations:** 1 Department of Life Sciences, Imperial College London, South Kensington SW7 2AZ, UK; 2 Department of Anesthesiology & Perioperative Medicine, Xijing Hospital, Xi’an, Shaanxi 710032, China; 3 Institute of Neuroinformatics, University of Zürich/ETH Zürich, Winterthurerstrasse 190, 8057 Zürich, Switzerland; 4 Centre of Excellence in Neurotechnology and UK Dementia Research Institute, Imperial College London, South Kensington SW7 2AZ, UK

**Keywords:** anesthetic mechanisms, neuronal circuitry, orexin, sedative, sleep architecture, tetanus toxin

## Abstract

The lateral habenula has been widely studied for its contribution in generating reward-related behaviors [1, 2]. We have found that this nucleus plays an unexpected role in the sedative actions of the general anesthetic propofol. The lateral habenula is a glutamatergic, excitatory hub that projects to multiple targets throughout the brain, including GABAergic and aminergic nuclei that control arousal [3, 4, 5]. When glutamate release from the lateral habenula in mice was genetically blocked, the ability of propofol to induce sedation was greatly diminished. In addition to this reduced sensitivity to propofol, blocking output from the lateral habenula caused natural non-rapid eye movement (NREM) sleep to become highly fragmented, especially during the rest (“lights on”) period. This fragmentation was largely reversed by the dual orexinergic antagonist almorexant. We conclude that the glutamatergic output from the lateral habenula is permissive for the sedative actions of propofol and is also necessary for the consolidation of natural sleep.

## Highlights

• Blocking the output of the LHb greatly diminishes the sedative effects of propofol
• Natural NREM sleep is also highly fragmented, particularly during “lights on”
• Stimulating the lateral habenula suppresses motor activity

## Teaser

Gelegen et al. show that the lateral habenula is a key hub whose excitation is permissive for the sedative effects of propofol. When the output from the lateral habenula is blocked, propofol’s sedative effects are greatly diminished, and NREM sleep is highly fragmented.

## Results and Discussion

Propofol is the most widely used intravenous (i.v.) general anesthetic, and its molecular target, the GABA_A_ receptor, has long been known [6, 7, 8]; however, the neuronal circuits that mediate its sedative and anesthetic effects are a mystery [9]. In humans and rodents, the effects of propofol on the electroencephalogram (EEG) have been thoroughly characterized, with the key feature being the increased coherence of thalamocortical oscillations [10, 11, 12], with changes in the higher-order thalamic nuclei correlating with loss of consciousness [10, 13]. But which neuronal circuits trigger these changes? Because low-frequency thalamocortical oscillations also increase during NREM sleep, an obvious possibility was that propofol was directly affecting hypothalamic nuclei involved in sleep and arousal [14, 15]. The arousal-promoting histaminergic neurons in the hypothalamic tuberomammillary nucleus have since been shown to be a plausible target for GABAergic sedatives, such as zolpidem [16, 17], and modulation of hypothalamic pathways has also been implicated in the actions of volatile general anesthetics [18, 19].

During the sedation produced by systemically administered GABAergic general anesthetics, as well as by the α2 adrenergic agonist dexmedetomidine, cFOS expression increases in sleep-promoting neurons and decreases in arousal-promoting neurons [14, 20, 21]. A comprehensive study [22] surveyed cFOS expression throughout the brain in response to a variety of sedatives and compared these changes with those observed during the natural sleep-wake cycle. One remarkable observation in this and a subsequent report [23] was that sedative agents induced marked cFOS expression in the lateral habenula (LHb). Because cFOS expression marks neuronal excitation, we have investigated whether the sedative actions of propofol require neurons of the LHb to be excited.

### Propofol Selectively Induces cFOS Expression in the LHb

**Figure 1:**
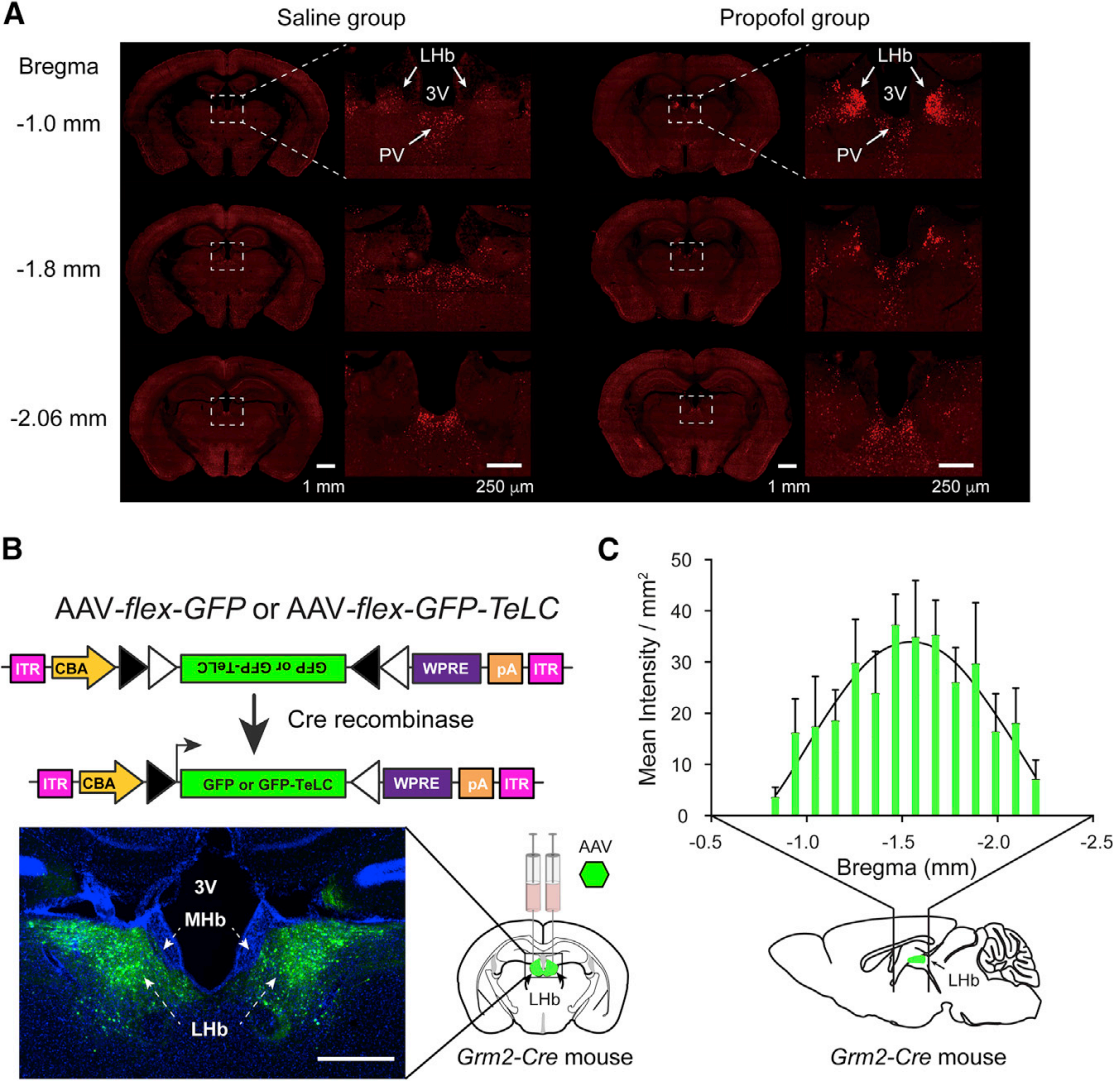
Propofol Induction of cFOS in the LHb and Experimental Design (A) The general anesthetic propofol induces marked expression of c-FOS in the LHb (n = 6) compared to saline injection (n = 4), showing propofol activates the LHb. The images on the left are representative brain sections stained for c-FOS after saline injection. The images on the right are representative brain sections stained for c-FOS after propofol injection. PV, paraventricular thalamic nucleus; 3V, third ventricle. (B) The output from the LHb can be silenced by expression of tetanus toxin light chain in LHb neurons using *Grm2-Cre* mice. Adeno-associated viral (AAV) constructs expressing either GFP (AAV-*flex-GFP*) or GFP-TeLC (AAV-*flex-GFP-TeLC*) in Cre-positive neurons were injected bilaterally into the LHb of *Grm2-Cre* mice. ITR, inverted terminal repeats; CBA, chicken beta-actin promoter/enhancer; LHb, lateral habenula; MHb, medial habenula; pA, polyadenylation signal; WPRE, woodchuck-postranscriptional-regulatory element; scale bar, 500 μm. (C) The extents of bilateral injections of AAV*-flex-GFP-TeLC* into the LHb of *Grm2-Cre* mice along the AP axis. The graph shows fluorescence intensity across the LHb for *GFP-TeLC* mice (means ± SEM; n = 19–32 sections from 4 mice). See also Figures S1–S3.

We first confirmed that sedative doses of propofol do indeed increase cFOS expression in the LHb above those induced by saline injection (previous studies had used a variety of GABAergic drugs but propofol has not itself been investigated in this regard). Basal cFOS expression was found throughout the brain, including in the neocortex and the midline thalamic nuclei (Figure 1A; left panels). Figure 1A (right panels) also shows that a sedative dose of propofol (212 ± 22 s of loss of righting reflex; mean ± SD; n = 6) caused a marked expression of cFOS in the LHb above the levels seen with a saline injection. We explored the possibility that propofol might directly excite LHb neurons but found that in acute slices from *LHb-GFP* mice, 1.5 μM propofol, an appropriate concentration for loss of righting reflex (LORR) [24], had no effect on the resting membrane potential (V_m_ = −46.2 ± 2.1 mV for control, V_m_ = −46.6 ± 2.4 mV with propofol; n = 15, p = 0.59; paired two-tailed t test), showing that the excitation must be by disinhibition elsewhere in the circuitry. To investigate whether or not this propofol-induced excitation (disinhibition) of the LHb was a cause or a consequence of sedation, we sought to block the glutamatergic output of the LHb to see whether this affected the sedative actions of the drug.

### Excitatory Output from the LHb Can Be Selectively Blocked Using *Grm2-Cre* Mice and Tetanus Toxin Light Chain

To selectively manipulate the LHb, we identified the metabotropic glutamate receptor 2-Cre recombinase (*Grm2-Cre*) mouse line, which has Cre recombinase expression in the LHb, but not the medial habenula (MHb) (see STAR Methods for a description of this mouse line). We then silenced the LHb with an adeno-associated virus (AAV) that expressed a Cre-dependent tetanus-toxin-light-chain (TeLC) transgene [25]. This toxin cleaves the vesicle-associated membrane protein synaptobrevin-2, which plays a key role in neurotransmitter release [26]. We injected AAV*-flex-GFP-TeLC* bilaterally into the LHb of *Grm2-Cre* mice to selectively block neurotransmission from these neurons (*LHb-TeLC* mice) (Figures 1B, S1, S2A, and S2B). Control mice were generated by bilaterally injecting AAV-*flex-GFP* into *Grm2-Cre* mice (*LHb-GFP* mice). *LHb-TeLC* mice had no overt neurological symptoms, and their weights (30.2 ± 4.3; mean ± SD) did not differ (p = 0.12; unpaired two-tailed t test) from *LHb-GFP* controls (33.2 ± 4.8; mean ± SD). The Cre-dependent transgene expression in cell bodies was confined to the LHb (Figures 1B and S1). There was no cell body expression in the MHb, or in midline thalamic structures. *GFP-TeLC* transgene expression was seen in *Grm2-Cre* neurons throughout the LHb. To further visualize axons from these neurons, we also injected AAV*-flex-ChR2-EYFP* (*LHb-ChR2* mice) (Figures S2C and S2D). We traced the projections from these neurons to their targets (Figures S2 and S3). Many axonal fibers in the *LHb-TeLC* and *LHb-ChR2* mice were strongly positive, including the *fasciculus retroflexus*, the main fiber bundle from the LHb (Figures S2 and S3). In addition to the anticipated projections to the ventral tegmental area (VTA), substantia nigra, and dorsal raphe areas (Figure S2), there were also unexpected GFP-TeLC- and ChR2-EYFP-positive fibers outlining the midline thalamic nuclei (including the central lateral, the central medial, the intermediodorsal, and the reunions nuclei), in the lateral hypothalamus, in the preoptic hypothalamus, especially the median preoptic nucleus, the septal hippocampal nucleus, the dorsal-lateral caudate-putamen (Figures S2 and S3), and in the prefrontal cortex (Figure S3), as well as in the mammillary area (Figure S2). Thus *Grm2-Cre* neurons in the LHb project more widely than anticipated, and their firing is likely to influence many brain regions.

To determine the transmitter phenotype, we patched EYFP-positive LHb neurons in acute slices from *LHb-ChR* mice, extracted their mRNA, and did real-time PCR assays (Figure S3A). EYFP-positive (*Grm2-Cre*) LHb neurons, and also non-EYFP-expressing LHb neurons, expressed the *Vglut2* gene, which encodes a glutamate vesicular transporter, but many cells also expressed low levels of the GABAergic *Gad1(Gad67)* gene (Figure S3). In a parallel assay to confirm specificity of the PCRs, neocortical pyramidal neurons randomly chosen and patched from the same slices did not contain *Vglut2* or *Gad1* transcripts (Figure S3). To confirm that *Grm2-Cre* LHb neurons used glutamate as their predominant neurotransmitter, and that GFP-TeLC blocked their transmitter release, we injected either AAV*-flex-ChR2-EYFP* alone into the LHb of *Grm2-Cre* mice (*LHb-ChR2* mice) or co-injected AAV*-flex-ChR2-EYFP* and AAV*-flex-GFP-TeLC* (*LHb-ChR2/LHb-TeLC* mice). We then made acute brain slices from several example projection regions, containing either prefrontal cortex (PFC) or dorsal caudate-putamen, from both groups of mice. In slices from these areas of *LHb-ChR2* mouse brains, light pulses evoked excitatory postsynaptic currents (EPSCs) (in 100% of cells), but not if the light pulses were given in the presence of the AMPA/NMDA receptor antagonists 6-cyano-7-nitroquinoxaline-2,3-dione (CNQX) and AP-5 (Figures S3F and S3G). Thus, these LHb neurons are glutamatergic. In slices from PFC and dorsal caudate-putamen of *LHb-ChR2/LHb-TeLC* mice, light stimulation evoked EPSCs in only about 35% of neurons (Figure S3H), presumably because the neurons that gave rise to these axons had not been co-transduced with the two AAVs. But overall, the large decrease in evoked EPSCs in *LHb-ChR2/LHb-TeLC* confirmed that TeLC expression in *Grm2-Cre* neurons in the LHb blocks their neurotransmitter release.

### Blocking Output from the LHb Greatly Diminishes the Sedative Effects of Propofol

**Figure 2:**
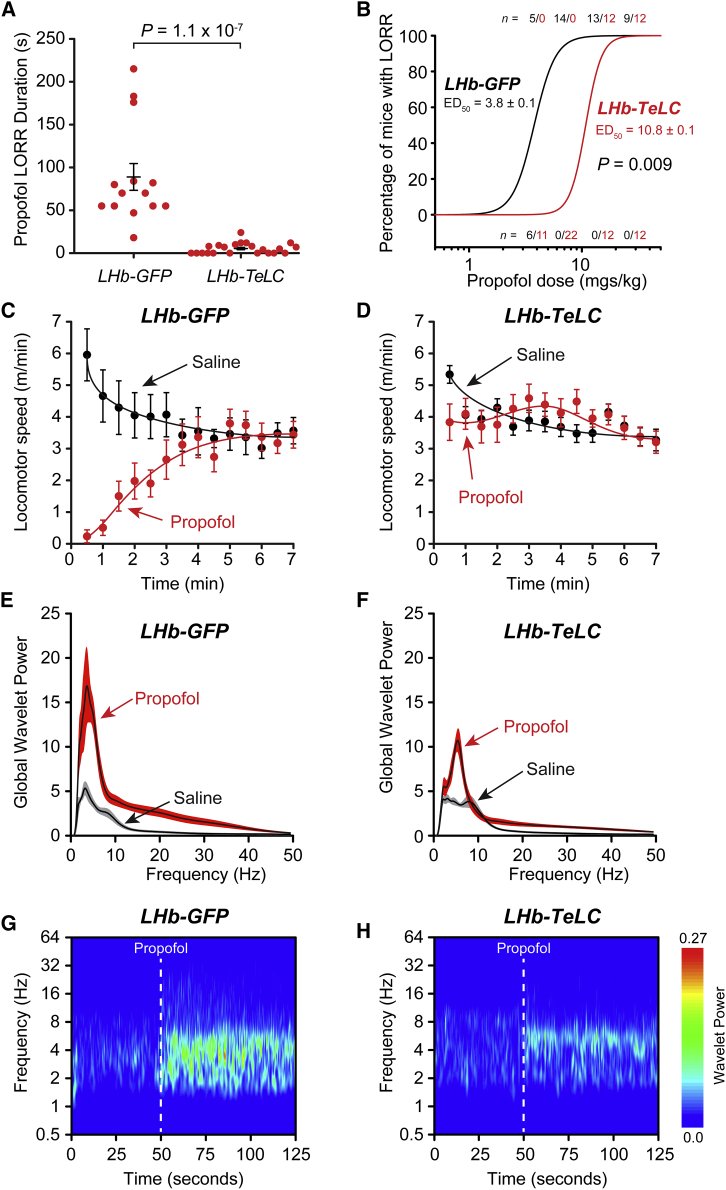
Silencing Output from *Grm2-Cre* Neurons in the LHb Reduces Propofol-Induced Sedation and LORR (A) Propofol-induced LORR at 7 mg/kg (i.v.) was blocked in *LHb-TeLC* mice (unpaired two-tail t test; p = 1.1 × 10^−7^, *t* = 6.69, df = 34, n = 14 *LHb-GFP* mice and 22 *LHb-TeLC* mice). (B) Propofol caused a nearly 3-fold rightward shift in the quantal dose-response curve (p = 0.009, unpaired two-tailed t test, *t* = 70, df = 1). (C) Propofol-induced (7 mg/kg) sedation in control *LHb-GFP* mice (two-way ANOVA; p < 1.0 × 10^−4^, F_17,414_ = 4.80, n = 11 saline and 14 propofol). (D) Lack of propofol-induced (7 mg/kg) sedation in *LHb-TeLC* mice (two-way ANOVA; p = 0.26, F_17,731_ = 1.19, n = 21 saline and 23 propofol). (E) Propofol-induced (7 mg/kg) changes in the global EEG wavelet power spectra from control *LHb-GFP* mice (p = 0.02, paired two-tailed t test, n = 7 saline and 7 propofol), (F) Propofol (7 mg/kg) induced much smaller changes in the global EEG wavelet power spectra in *LHb-TeLC* mice (p = 0.03, paired two-tailed t test, n = 7 saline and 7 propofol). (G) The effects of propofol on the average wavelet power spectrum for *LHb-GFP* mice (n = 7) showing the large increases in EEG power. Propofol was injected at 50 s. (H) The effects of propofol on the average wavelet power spectrum for *LHb-TeLC* mice (n = 7) showing the much smaller increases in EEG power. Propofol was injected at 50 s. Symbols in (A), (C), and (D) are means ± SEMs. (E and F) Lines and error envelopes represent the mean and SEM, respectively.

We next investigated the effects of bolus doses of propofol sufficient to induce sedation and LORR, but not deep anesthesia. At a dose of 7 mg/kg (i.v.) *LHb-GFP* mice had LORR that lasted on average for 90 ± 16 s (mean ± SEM; n = 14) (Figure 2A). LHb*-TeLC* mice by contrast were virtually unresponsive to this dose of propofol: they had only a short (5 ± 1 s; n = 22) LORR (Figure 2A). The quantal dose-response curve for propofol (Figure 2B) was shifted to the right by about a factor of 3. Even after they recovered from LORR, the locomotion of the *LHb-GFP* mice was impaired (p < 10^−4^) for several minutes following propofol injection compared with saline injected controls (Figure 2C), whereas the locomotion of the *LHb-TeLC mice* was unaffected (p > 0.3) (Figure 2D). These differences in propofol sensitivity were reflected in the EEG: in *LHb-GFP* control mice, a propofol injection compared with saline elicited a nearly 3-fold increase in power, with increases in both delta frequency power, as well as producing an increase in the power of a broad range of frequencies >10 Hz extending to the gamma range, 30–40 Hz (Figure 2E), as we found in rats [10]. By contrast, in *LHb-TeLC* mice, propofol injection, when compared with a saline injection, produced much smaller (∼60% increase) changes in delta frequency power, as well as at higher frequencies (Figure 2F), although the overall increase in power was still significant (p = 0.034). Wavelet spectra as a function of time (Figures 2G and 2H) show that these increases in EEG power occur almost immediately following propofol injection (at 50 s).

### Stimulating Output from the LHb Reduces Motor Activity

**Figure 3:**
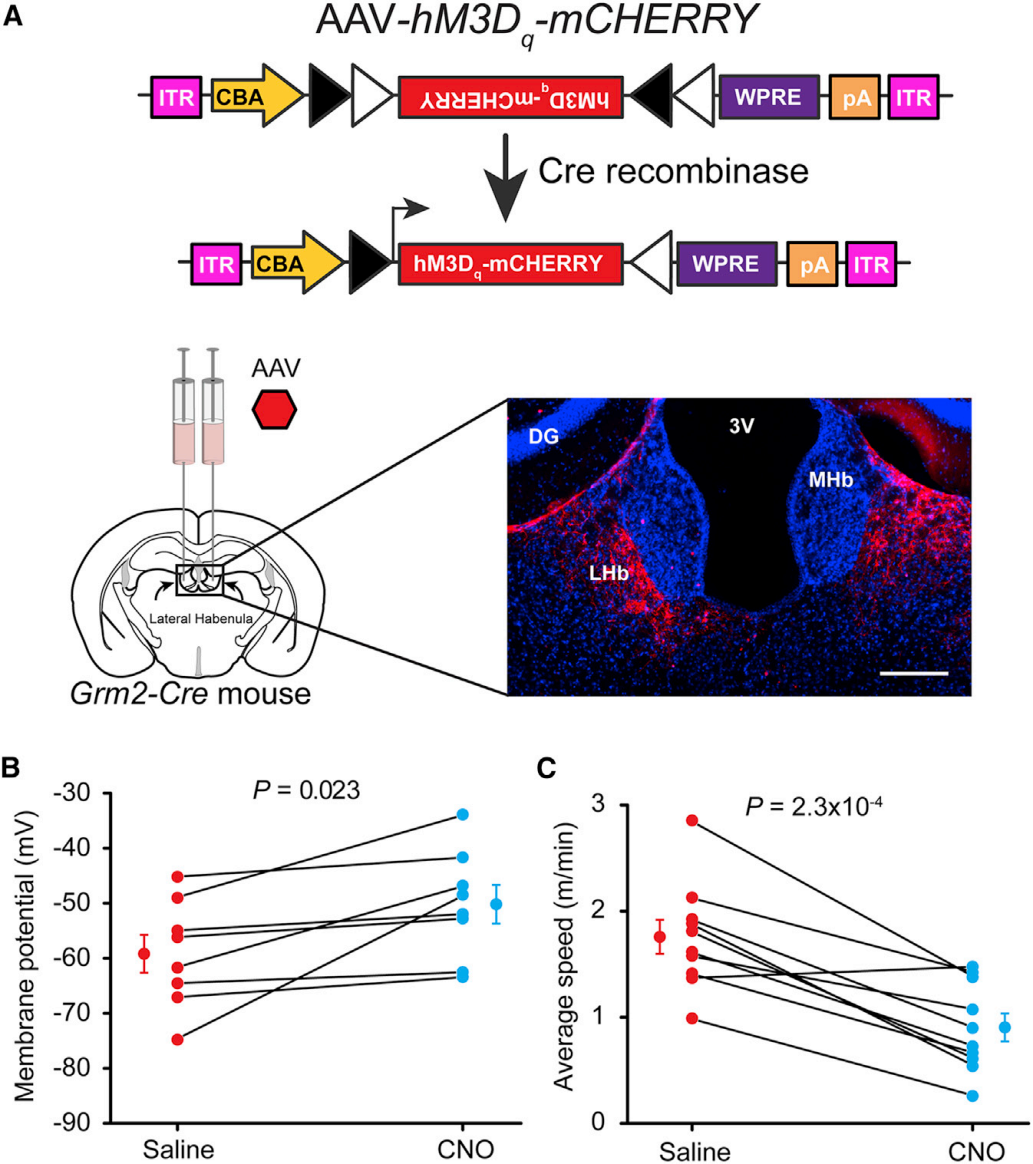
Pharmacogenetic Stimulation of *Grm2-Cre* LHb Neurons Reduces Locomotor Activity (A) Bilateral injection of AAV*-flex-hM3D*_*q*_*-mCHERRY* into the LHb region of *Grm2-Cre* mice; scale bar, 200 μm. Expression of the receptor, detected with mCherry immunocytochemistry, was restricted to cells in the LHb. DG, dentate granule cells; 3V, third ventricle. (B) Bath application of 5 μM CNO in a brain slice preparation led to an increase in resting membrane potential of 9.0 ± 3.1 mV from −59.2 ± 3.5 to −50.2 ± 3.5 mV (paired two-tailed t test; n = 8 neurons, 3 mice; p = 0.023, *t* = 2.88, df = 7). Resting membrane potential was calculated as an average voltage (sampled every 200 ms) between 1 and 3 min immediately before bath application of CNO and at the peak of the effect 5–10 min after drug administration. (C) Locomotor speed was recorded in an open field for 20 min, 20 min after CNO (5 mg/kg, i.p.) injection or saline injection, and the speed was reduced approximately 2-fold (paired two-tailed t test; n = 10 mice; p = 2.26 × 10^−4^, *t* = 5.91, df = 9) following CNO injection. (B and C) Symbols are mean ± SEM.

Acute electrical stimulation of the LHb in cats strongly induces NREM sleep [27], yet on the other hand, lesioning the LHb in rats slightly decreases the amount of REM sleep and theta power in the EEG without affecting NREM sleep [28]. There are, however, many subtypes of glutamatergic projection neuron in the LHb [29, 30]; for example, only subsets of LHb neurons convey error prediction [1]. Similarly, it is feasible that only certain neuronal subtypes in the LHb are responding to propofol. To corroborate our results obtained with propofol, we tested whether pharmacogenetic excitation of *Grm2-Cre* neurons in the LHb mimicked the effects of propofol. We bilaterally injected AAV*-flex-hM3D*_*q*_*-mCHERRY* into the LHb of *Grm2-Cre* mice (*LHb-hM3D*_*q*_ mice, Figure 3A); hM3D_q_ receptor expression was confined to the LHb (Figure 3A). The metabotropic hM3D_q_ receptor, when activated by its ligand clozapine-N-oxide (CNO), is excitatory [31]. In acute slices containing LHb, we patch-clamped neurons expressing the hM3D_q_ receptor (identified by mCHERRY fluorescence). CNO application caused a gradual depolarization (9.0 ± 3.1 mV; n = 8) (Figure 3B), often resulting in a train of action potentials. Systemically injecting *LHb-hM3D*_*q*_ mice with CNO decreased their movement ∼2-fold (p = 2.3 × 10^−4^; n = 10) (Figure 3C) compared with saline injections. During the time that this reduction in mobility was recorded (20–40 min after CNO injection), there was no significant increase in the percentage of time scored as NREM sleep (p = 0.52). Because CNO is metabolized to clozapine, which also acts as a ligand at hM3D_q_ receptors [32], we checked that CNO (5 mg/kg i.p.) did not significantly affect motor activity in *LHb-mCherry* mice compared to saline injection (p = 0.44, two-tailed paired t test n = 5).

### Blocking Output from the LHb Causes Marked Fragmentation of NREM Sleep

**Figure 4:**
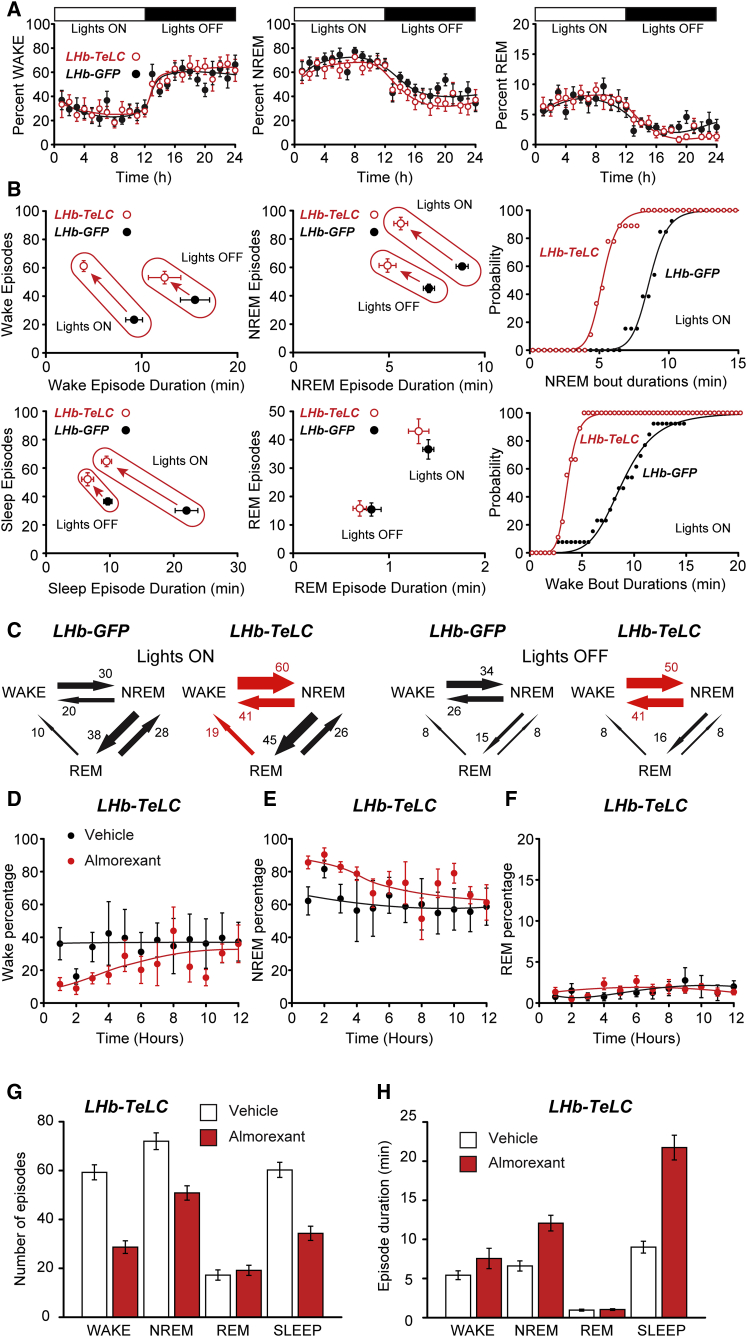
Silencing Output from *Grm2-Cre* Neurons in the LHb Causes Sleep Fragmentation (A) Percentage of time during wake, NREM, and REM was identical between *LHb-GFP* and *LHb-TeLC* mice (two-way ANOVA; wake: p = 0.96, F_23,456_ = 0.53; NREM: p = 0.96, F_23,456_ = 0.53; REM: p = 0.84, F_23,456_ = 0.71. (B) Four left-hand panels: the number of episodes of wake, NREM, and sleep (defined as a consolidated period of NREM and REM) was larger in *LHb-TeLC* mice (n = 9) than *Lhb-GFP* mice (n = 13), although their durations were generally proportionately shorter (unpaired two-tail t tests). These changes were greater during “lights ON” (wake episodes: p = 6.7 × 10^−9^, *t* = 9.56; wake duration: p = 9.3 × 10^−5^, *t* = 4.87; NREM episodes: p = 2.2 × 10^−6^, *t* = 6.54; NREM duration: p = 1.7 × 10^−6^, *t* = 6.66; sleep episodes: p = 3.5 × 10^−8^, *t* = 8.63; sleep duration: p = 1.8 × 10^−5^, *t* = 5.58; df = 20) compared with “lights OFF” (wake episodes: p = 1.7 × 10^−3^, *t* = 3.63; wake duration: p = 0.18, *t* = 1.38; NREM episodes: p = 6.4 × 10^−3^, *t* = 3.05; NREM duration: p = 3.9 × 10^−4^, *t* = 4.26; sleep episodes: p = 3.6 × 10^−3^, *t* = 3.29; sleep duration: p = 7.8 × 10^−3^, *t* = 2.95; df = 20). There were no changes (unpaired two-tail t test) in either episode number or duration for REM for either “lights ON” (p = 0.26, *t* = 1.17 and p = 0.38, *t* = 0.09, df = 20) or “lights OFF” (p = 0.91, *t* = 0.11 and p = 0.38, *t* = 0.90, df = 20). Two right-hand panels: cumulative distributions for wake and NREM bout durations during “lights ON.” (C) Blocking the output of LHb *Grm2-Cre* neurons with TeLC greatly increased wake-NREM and NREM-wake transitions (red arrows) during both “lights ON” (p = 7.0 × 10^−6^, *t* = 5.92 and p = 1.4 × 10^−4^, *t* = 4.62, respectively; df = 21) and “lights OFF” (p = 0.01, *t* = 2.58 and p = 8.0 × 10^−3^, t = 2.92, respectively, df = 21) but also caused an increase in REM-wake transitions during “lights ON” (p = 7.0 × 10^−3^, *t* = 2.98, df = 21, red arrow). The values by the arrows are average transitions per hour. (D–F) The orexin receptor antagonist, almorexant, largely reversed the sleep fragmentation that was observed in the *LHb-TeLC* mice. During the 12 hr after i.p. injection of almorexant (30 mg/kg) into *LHb-TeLC mice*, (D) the percentage of time in the wake state was reduced (repeated-measures ANOVA, *F*_*treatment*_*(1,96) = 8.27, p = 0.005*), (E) the percentage of time in the NREM state was increased (repeated-measures ANOVA, *F*_*treatment*_*(1,96) = 9.45, p = 0.003*), but (F) there was no change in the time spent in REM. (G) The number of wake (p = 1.4 × 10^−4^), NREM (p = 0.059), and sleep (consolidated NREM + REM) episodes (p = 0.002) were reduced, with no change in the number of REM episodes. (H) Moreover, the average durations of wake, NREM (p = 0.035), and sleep (p = 2.1 × 10^−4^) increased, with no change in the average duration of REM episodes (n = 4 vehicle and n = 6 almorexant). Where error bars are shown they represent SEM. See also Figure S4.

Because manipulations of anesthetic targets often affect sleep architecture [16, 33], we next explored whether chronically silencing LHb *Grm2-Cre* neurons affected natural sleep over 24 hr (Figure 4). *LHb-TeLC* mice did not differ from *LHb-GFP* mice in their overall time spent in wake, NREM, or REM sleep (p > 0.8; n = 9–11); both groups of mice had a typical 24-hr activity profile, with less wakefulness during “lights ON” (Figure 4A), although the delta power during NREM in *LHb-TeLC* mice was slightly lower than that in *LHb-GFP* mice (p = 0.02) (Figure S4A). However, there was a large difference in sleep consolidation (Figures 4B, S4B, and S4C). The number of wake and NREM episodes was substantially higher in *LHb-TeLC* mice compared with *LHb-GFP* mice, particularly during “lights ON,” but the durations of these states were proportionately reduced (Figures 4B, S4B, and S4C). Taking this analysis further, the duration of entire sleep episodes (defined as REM plus NREM in a continuous block) decreased strongly, particularly during “lights ON,” but their number increased proportionately (Figures 4B, S4B, and S4C). REM sleep frequency and duration was unchanged in *LHb-TeLC* mice compared with *LHb-GFP* mice, but in *LHb-TeLC* mice, there was a high number of REM-wake transitions that were not apparent in control mice (Figure 4C). Thus, *LHb-TeLC* mice had a severely fragmented pattern of natural NREM sleep.

Why should the sleep fragmentation phenotype be greatest during the “lights-ON” period? The LHb receives afferents from both the suprachiasmatic nucleus that houses the master circadian clock and the pineal gland [3]. LHb neurons fire when light activates the retina [34] but also maintain an intrinsic circadian rhythmicity in action potential firing in acute brain slices [34, 35]. Thus, LHb activity could help maintain sleep during daylight, when mice are resting more. Intriguingly, we found that the sleep fragmentation phenotype of *LHb-TeLC* mice was largely reversed by systemic administration of the dual orexin receptor antagonist almorexant (Figures 4D–4H). Almorexant caused an overall reduction in waking that lasted several hours (Figure 4D) and a corresponding increase in NREM sleep (Figure 4E), with no significant changes in REM (Figure 4F). These changes were due to the number of wake and sleep (consolidated NREM + REM) episodes decreasing, but the durations of NREM and sleep increasing (Figures 4G and 4H). These results suggest that excessive activation of the orexin system contributes to the sleep-wake fragmentation in *LHb-TeLC* mice. This is perhaps because of failure to excite GABA neurons that inhibit orexin neurons and is consistent with the presence of LHb *Grm2-Cre* axons in the lateral hypothalamic area (Figure S2). This explanation is consistent with the observation that in mice that overexpress orexin [36], sleep is fragmented with significantly more wake and NREM episodes, but with reduced durations, particularly during “lights ON.”

### What Role Does the Propofol-Induced Excitation of the LHb Play in Sedation?

It has been hypothesized that the LHb has diverse roles, all united by a common theme of motor suppression [3]. The LHb is a glutamatergic hub [3, 5] that receives input from diverse forebrain regions (e.g., the PFC, basal ganglia, and preoptic and lateral hypothalamus [3, 5]) and projects to GABAergic neurons of the rostromedial tegmental nucleus [37], a nucleus at the caudal end of the VTA. *Trans*-synaptic retrograde tracing has shown that GABAergic neurons throughout the VTA receive a disproportionately large input from the LHb [38], and this could provide a powerful inhibitory control of motor responses [4] by inhibiting dopamine and serotonin neurons [2, 3]. In keeping with its diverse modulatory roles, dopamine also supports wakefulness [39]. Selectively activating dopamine neurons optogenetically in a downstream target of the LHb, the VTA, induces both consolidated wakefulness [39] and also prompt wakening from general anesthesia [40].

The above findings are certainly consistent with our observation that selective excitation of the LHb reduces motor activity. Because blocking LHb glutamatergic output prevents propofol-induced loss of muscle tone (LORR) and also reduces the propofol-induced enhancement in EEG power, this implies the LHb must be able to modulate the thalamocortical coherence that is a hallmark of propofol’s sedative and anesthetic effects. It is most commonly assumed that sedation is a consequence of anesthetics activating, or potentiating, inhibitory circuits. Our findings, however, show that the activation of an excitatory pathway is mechanistically essential for propofol-induced sedation.

## STAR★Methods

### Key Resources Table

**Table:** 

REAGENT or RESOURCE	SOURCE	IDENTIFIER
**Antibodies**		

Anti-EGFP rabbit polyclonal antibody	Thermo Fisher Scientific	A6455; RRID: AB_221570
Anti-mCherry mouse monoclonal antibody	Clontech	632543; RRID: AB_2307319
Anti-cFos rabbit polyclonal antibody	Synaptic System	226003; RRID: AB_2231974
Alexa Fluor 488 goat anti-rabbit IgG	Molecular Probes	A11034; RRID: AB_2576217
Alexa Fluor 594 goat anti-mouse IgG	Molecular Probes	A11005; RRID: AB_141372
Alexa Fluor 594 donkey anti-rabbit IgG	Life Technologies	ab150076; RRID: AB_2340621
Alexa Fluor 555 streptavidin Conjugate	Thermo Fisher Scientific	S32355; RRID: AB_2571525

**Bacterial and Virus Strains**		

AAV*-flex-GFP-TeLC*	[41]	N/A
AAV*-flex-EGFP*	This paper	N/A
AAV*-flex-ChR2*(*H134R*)*-EYFP*	This paper	N/A
AAV*-flex-hM3D*_*q*_*-mCHERRY*	[42]	N/A

**Chemicals, Peptides, and Recombinant Proteins**		

Sodium deoxycholate	Sigma-Aldrich	D5670
Benzonase endonuclease	Sigma-Aldrich	E1014
Isoflurane	Zoetis	50019100
Injectable Propofol	Fresenius Kabi	https://www.fresenius-kabi.com/fi/documents/SmPC_Propofol_2__MCT.pdf
Clozapine *N*-Oxide	Tocris	4936
Pentobarbital Sodium solution	JML	M042
Paraformaldehyde 16%	Alfa Aesar	30525-89-4
Triton X-100	Sigma-Aldrich	T8787
Normal goat serum	Vector Laboratories	S-1000
2,6-Diisopropylphenol	Aldrich	D12660-8
CNQX	Tocris	0190
D-AP5	Tocris	0106
Biocytin	Sigma	B4261

**Critical Commercial Assays**		

Single Cell-to-CT Kit for qRT-PCR	Thermo Fisher Scientific	4458236

**Experimental Models: Cell Lines**		

HEK293 cells	Sigma-Aldrich	85120602; RRID: CVCL_0045

**Experimental Models: Organisms/Strains**		

*Tg*(*Grm2-cre*)*MR90Gsat/Mmcd*	GENSAT	MR90-CRE

**Oligonucleotides**		

*Grm2*(*34611*) *F* 5′-GGCAGCCACTCTTTGGTTC TACTC-3′	MMRRC	034611-UCD
*CreGS-R1* 5′-CGGCAAACGGACAGAAGCATT-3′	MMRRC	034611-UCD
*Vglut2/solute carrier family 17* gene expression assays	Thermo Fisher Scientific	Mm00499876_m1
*Gad67/glutamate decarboxylase* gene expression assays	Thermo Fisher Scientific	Mm04207432_g1
18S ribosomal RNA gene expression assays	Thermo Fisher Scientific	Mm04277571_s1

**Recombinant DNA**		

pAAV-FLEX-GFP plasmid	Addgene	28304
pAAV-EF1a-double floxed-hChR2(H134R)-EYFP-WPRE-HGHpA	Addgene	20298
pAAV-hSyn-DIO-hM3D(Gq)-mCherry	Addgene	44361
Adenovirus helper plasmid *pFΔ6*	Donated by M Klugmann [43]	N/A
AAV helper plasmid *pH21* (AAV1)	Donated by M Klugmann [43]	N/A
AAV helper plasmid *pRVI* (AAV2)	Donated by M Klugmann [43]	N/A

**Software and Algorithms**		

Spike2	Cambridge Electronic Design	http://ced.co.uk/products/spkovin
MATLAB	MathWorks	https://uk.mathworks.com/
Activity Monitor Version 5 for mice	Medical Associates	http://www.med-associates.com/product-category/activity-software/
WinWCP, Version 4.1.2	Strathclyde Electrophysiology Software	http://spider.science.strath.ac.uk/sipbs/showPage.php?page=software_ses
WinEDR, Version 3.0.9	Strathclyde Electrophysiology Software	http://spider.science.strath.ac.uk/sipbs/showPage.php?page=software_ses
SDS 2.1	Thermo Fisher Scientific	https://www.thermofisher.com/uk/en/
Prism6	GraphPad Software	https://www.graphpad.com
Origin	OriginLab	https://www.originlab.com/

**Other**		

1-ml HiTrap Heparin column	Sigma-Aldrich	5-4836
Amicon Ultra-4	Millipore	UFC810024
Angle Two stereotaxic frame	Leica Microsystems	N/A
Hamilton microliter 10-μl syringes	Hamilton	701
Custom made 33-gauge stainless steel needle	Hamilton	7803-05
Borosilicate glass capilaries	Harvard Apparatus	GC150F-10
Neurologger 2A	[44, 45]	N/A
Vibratome	Leica Microsystems	VT1000S
Vibratome tissue slicer	Campden Instruments	7000smz
Blue (470 nm) collimated LED	Thorelabs	M470L3-C1
StepOnePlus Real-Time PCR Systems	Thermo Fisher Scientific	4376600
Uplight Microscope	Olympus	BX51W1
Upright Microscope	Scientifica	S-Scope
SciCam Pro	Scientifica	100918
Four wavelength high power LED source	Thorelabs	LED4D
Multiclamp 700B amplifier	Molecular Devices	N/A
DAD	National Instruments	BND-2100

### Contact for Reagent and Resource Sharing

Further information and requests for resources and reagents should be directed to and will be fulfilled by the Lead Contact, Nicholas P. Franks (n.franks@imperial.ac.uk).

### Experimental Model and Subject Details

#### Mice

All experiments were performed in accordance with the United Kingdom Home Office Animal Procedures Act (1986), and had local ethical approval. The *Grm2-Cre* mouse line was generated by GENSAT and obtained from the Mouse Mutant Resource Center (UC Davis, Davis, CA), stock *Tg*(*Grm2-cre*)*MR90Gsat/Mmcd* (The Gene Expression Nervous System Atlas - GENSAT - Project, NINDS Contracts N01NS02331 & HHSN271200723701C to The Rockefeller University, New York, NY). The line was generated by pronuclear injection of a BAC transgene containing a Cre recombinase reading frame inserted into the metabotropic glutamate receptor 2 gene [41]. We maintained the line as heterozygotes. Genotyping primers for the *Grm2-Cre* line were *Grm2*(*34611*)*F* (5′-GGCAGCCACTCTTTGGTTCTACTC-3′) and *CreGS-R1* (5′-CGGCAAACGGACAGAAGCATT-3′); a 375 bp product indicated the transgene (protocol and primer sequences recommended by the Mouse Mutant Resource Centre, https://www.mmrrc.org/). Male mice (3 - 5 months old) were kept on a 12:12 light:dark cycle, 22 ± 1°C, 50% relative humidity, at a maximum of four animals per cage, with free access to food and water. Following surgery, mice were kept singly housed. Behavioral experiments, except where specified otherwise, were performed during the “Lights OFF” period.

### Method Details

#### AAV transgenes

The AAV*-flex-GFP-TeLC* transgene plasmid was described previously [25]. The GFP protein is fused to the N terminus of TeLC. The AAV*-flex-EGFP* transgene was Addgene plasmid 28304 (gift from Edward Boyden, MIT, Cambridge, USA). The AAV*-flex-ChR2*(*H134R*)*-EYFP* transgene was a gift from Karl Deisseroth (Addgene plasmid 20298). This expresses the humanized ChR2 gene with histidine 134 changed to arginine, to make larger currents; EYFP is fused to the C terminus of ChR2, which also makes it a good substrate for axonal transport. The AAV*-flex-hM3D*_*q*_*-mCHERRY* transgene was a gift from Bryan L. Roth (Addgene plasmid 44361) [42]. The mCherry protein is fused to the C terminus of hM3Dq.

#### Generation of recombinant AAV particles

All AAV transgenes were packaged into AAV capsids (mixed serotype 1 & 2, 1:1 ratio of AAV1 and AAV2 capsid proteins with AAV2 ITRs) [43]. HEK293 cells (obtained from the European Collection of Cell Cultures (ECACC) via Sigma-Aldrich) were co-transfected, using the calcium phosphate method, with AAV transgene plasmid, the adenovirus helper plasmid *pFΔ6*, and the AAV helper plasmids *pH21* (AAV1), and *pRVI* (AAV2) [43]. 60–65 hours after transfection, cells were washed in 1 x PBS, and pelleted; pellets were resuspended in 150 mM NaCl, 20 mM Tris pH 8.0. Then sodium deoxycholate (Sigma #D5670) and benzonase endonuclease (Sigma #E1014) were added and incubated at 37°C for 1 hr. After incubating, cell debris were removed by centrifugation and AAV particles were purified from the supernatant by passing over a heparin column (1 mL HiTrap Heparin columns, Sigma #5-4836), which binds the AAV particles. The column was pre-equilibrated with 10 mL 150 mM NaCl, 20 mM Tris pH 8.0. Then the supernatant was loaded onto the column at a flow rate of 200 μl/min; the column was washed with 20 mL 100 mM NaCl, 20 mM Tris pH 8.0 and virus was eluted off the column as follows: 1 mL 200 mM NaCl, 20 mM Tris pH 8.0 (discarded), 1 mL 300 mM NaCl, 20 mM Tris pH 8.0 (discarded), 1.5 mL 400 mM NaCl, 20 mM Tris pH 8.0 (collected), 3 mL 450 mM NaCl, 20 mM Tris pH 8.0 (collected), 1.5 mL 500 mM NaCl, 20 mM Tris pH 8.0 (collected). After purification, AAV particles were concentrated using Amicon Ultra-4 (100000MWCO, #UFC810024, Millipore, Watford, Hertfordshire, UK) at 2000 g for 10 min. The concentrator was twice refilled with 3.5 mL of 0.9% NaCl. Elutions were removed to a sterile tube, and 250 μL of 0.9% NaCl were added. AAV was aliquoted and stored at –80°C.

#### Stereotaxic injections of AAV

All the AAV-injection experiments used adult male heterozygote *Grm2-Cre* mice, 8–12 weeks old. Mice were anesthetized with 2% isoflurane in oxygen by inhalation and mounted into a stereotaxic frame (Angle Two, Leica Microsystems, Milton Keynes, Buckinghamshire, UK). AAV was injected using Hamilton microliter #701 10 μL syringes with a 33-gauge stainless steel needle (Point style 3, length 1.5 cm, Hamilton), back loaded with mineral oil and AAV mixture (1:1 with 20% mannitol) in the tip. For the *LHb-ChR2/LHb-TeLC* mice, the two AAVs, AAV*-flex-ChR2-EYFP* and AAV*-flex-TeLC-EGFP* were mixed 1:1 prior to injection. The coordinates of the (bilateral) injection sites according to the digital atlas of the Leica apparatus were relative to Bregma: AP, −1.70; ML -/+ 0.44; DV was consecutive, starting +2.90 (1/3 volume), +2.85 (1/3 volume), +2.80 (1/3 volume). To make the *LHb-ChR2/LHb-TeLC* mice, a total volume of 1 μL of AAV was divided into three aliquots for each side of the brain; to generate the *LHb-hM3Dq* mice, we used a total of 0.6 μL per brain side, again divided into three aliquots for each consecutive injection. Mice that had been injected with AAVs were allowed 1 month to recover in their home cages and for the viral transgenes to adequately express before being fitted with Neurologger 2A devices (see below) and undergoing behavioral experiments.

#### EEG and EMG recordings and sleep scoring

For non-tethered EMG and EEG recordings, mice were chronically implanted with skull screw electrodes (–1.5 mm Bregma, +1.5 mm midline – first recording electrode; +1.5 mm Bregma, –1.5 mm midline – second recording electrode; –1 mm Lambda, 0 mm midline – reference electrode) to measure cortical EEG. A pair of stainless steel EMG electrodes was implanted in dorsal neck muscle. The electrical signals were recorded on a wireless electronic recording device (Neurologger 2A) as described previously [44, 45]. Four data channels could be recorded at a sampling rate of 200 Hz and waveforms visualized using Spike2 software (Cambridge Electronic Design, Cambridge, UK) or MATLAB (MathWorks, Cambridge, UK). The EEG was high-pass filtered (1 Hz, −3dB) using a digital filter and the EMG was band-pass filtered between 5-48 Hz (−3dB). Power in the delta (0-4 Hz) and theta (6-10 Hz) bands were calculated, together with the RMS value of the EMG signal (averaged over a bin size of 5 s), and these were used to define the vigilance states of Wake, NREM and REM [46]. EEG data were analyzed using Fourier transforms to average power spectra over blocks of time. The Fourier transform power spectra were normalized such that the total area under the spectra for the saline controls was unity.

#### Assay for sedation

Propofol (Fresenius Kabi, Runcorn, Cheshire, UK), was delivered via tail-vein injection (i.v.). CNO (5 mg/kg, Cat. No. 4936, Tocris, Avonmouth, Bristol, UK) was administered by intraperitoneal injection (i.p.). For propofol-induced sedation, animals, fitted with Neurologger 2A devices, were placed immediately after i.v. injection in an activity cage to assess locomotor activity. For CNO experiments, mice were placed in the activity cages 20 minutes after i.p CNO injections (Activity Monitor Version 5 for mice, Medical Associates, St. Albans, VT). All mice were videoed. The EEG of animals was simultaneously recorded (see above).

#### Assay for LORR

Propofol was delivered via tail-vein injection and animals, fitted with Neurologger 2A devices [44], were tested for loss of righting reflex (LORR) by placing them gently on their backs [45]. Animals were scored as positive for LORR if they made no obvious attempt to right themselves. All mice were videoed. The EEG of animals was simultaneously recorded using Neurologger 2A devices [44, 45] (see above).

#### Immunohistochemistry

Under deep pentobarbital anesthesia (100 mg/kg body weight; *i.p.*), mice were fixed by transcardial perfusion with 4% paraformaldehyde in PBS, pH 7.4. Brains were removed and 35-μm-thick coronal sections cut using a Leica VT1000S vibratome. Free-floating sections were washed in PBS three times for 10 minutes, permeabilized in PBS plus 0.4% Triton X-100 for 30 min, blocked by incubation in PBS plus 4% normal goat serum (NGS), 0.2% Triton X-100 for 1 h (all at room temperature) and subsequently incubated with an EGFP antibody (, rabbit, 1:1000, A6455, Thermo Fisher Scientific, Loughborough, Leicestershire, UK) and/or a mCHERRY monoclonal antibody (mouse, 1:2000, #632543, Clontech, Mountain View, CA). Primary antisera were diluted in PBS plus 2% NGS (normal goat serum) overnight at 4°C. The next day, incubated slices were washed three times (each lasting 10 minutes), in PBS and then incubated for 2 h at room temperature in PBS plus 1% NGS with a 1:1000 dilution of a Alexa Fluor 488 goat anti-rabbit IgG (H+L) (1:1000, A11034, Molecular Probes, Eugene, OR) or Alexa Fluor 594 goat anti-mouse IgG (H+L) (1:1000, A11005, Molecular Probes), and subsequently washed there times in PBS for 10 min at room temperature. The sections were mounted on slides and coverslipped. For cFOS expression the primary antibody was cFOS (rabbit, 1:1000, Cat. No. 226003, Synaptic System), the secondary antibody was Alexa Fluor 594 donkey anti-rabbit IgG (1:1000, ab150076, Life Technologies).

#### Electrophysiology

Adult (3-6 months postnatal) mice were used, and brain slices were then prepared following cervical dislocation (in accordance with UK Home Office guidelines). The brain was rapidly removed from the skull after decapitation and immersed in ice-cold slicing solution composed of (in mM): 85 NaCl, 2.5 KCl, 1 CaCl_2_, 4 MgCl_2_, 1.25 NaH_2_PO_4_, 26 NaHCO_3_, 75 sucrose, 25 glucose, pH 7.4 when bubbled with 95%O_2_/5%CO_2_. The cerebellum was removed and the remaining forebrain was then glued to the center of the vibratome stage with the surface of the cut facing down. Coronal brain slices (250 μm thickness) were cut with a vibratome tissue slicer (Campden Instruments, Loughborough, Leicestershire, UK) and immediately transferred to a holding chamber containing slicing artificial cerebral spinal fluid (ACSF) bubbled with 95%O_2_/5%CO_2_. Once slicing was complete the holding chamber was then transferred to a 37°C heat block for 10 min, after which the slicing solution was exchanged for recording ACSF (in mM: NaCl 125, KCl 2.5, CaCl_2_ 2, MgCl_2_ 1, NaH_2_PO_4_ 1.25, NaHCO_3_ 26, glucose 11, pH 7.4 when bubbled with 95%O_2_/5%CO_2_). The slices were incubated in the recording ACSF at room temperature for at least another 20 minutes prior to electrophysiological recordings. For whole-cell current-clamp recordings, the internal solution contained the following (in mM): 145 K-gluconate, 4 NaCl, 5 KCl, 0.5 CaCl_2_, 5 EGTA, 10 HEPES, 4 Mg-ATP, and 5 sucrose, pH 7.3, adjusted with KOH. Whole-cell recordings were performed with a Multiclamp 700B amplifier (Molecular Devices, Wokingham, West Berkshire, UK). The analog output was low-pass filtered at 10 kHz and digitized at 20 kHz. Data acquisition was performed using WinWCP (Version 4.1.2) and WinEDR (Version 3.0.9) kindly provided by John Dempster (University of Strathclyde, UK). For optogenetic stimulation, a blue (470 nm) collimated LED (M470L3-C1, Thorlabs, Ely, Cambridgeshire, UK) was mounted to the back of the Olympus BX51W1 microscope and was controlled by self-programmed stimulus protocols in WinEDR. For the acute slices containing the PFC and caudate putamen in the *LHb-ChR2* and *LHb-ChR2/LHb-TeLC* mice, we patched cells surrounded by a high density of ChR2-EYFP-positive fibers when viewed under primary fluorescence.

### Single cell RT-PCR

Slices were transferred to a submersion recording chamber and were continuously perfused with fully oxygenated aCSF at room temperature. Neurons were visualized using infra-red LED under an upright microscope (S-Scope, Scientifica, UK) equipped with a 60x water immersion objective (1.0 numerical aperture) and a charge coupled device (CCD) video camera (SciCam Pro, Scientifica, UK). *Grm2-Cre* EYFP-positive neurons were identified by their EYFP signal under fluorescence illumination (LED4D, Thorlabs, coupled to YFP excitation filter). Borosilicate glass capillaries (1.5mm OD, 0.86mm ID, Harvard Apparatus, #GC150F-10) were autoclaved prior to pulling patching pipettes. Whole-cell patching was performed with a Multiclamp 700B amplifier (Molecular Devices, CA) using glass microelectrodes (4–6 MΩ in resistance) filled with RNase-free intracellular solution containing (in mM): 140 K-gluconate, 5 NaCl, 10 HEPES, 0.1 EGTA, 2 MgCl_2_, 2 Mg-ATP, and 0.3 Na-GTP (pH 7.3, osmolality 285 mOsm). 0.2% Biocytin was included in the intracellular solution to identify the cell position and morphology. For RNA extraction, 0.5-1μl of intracellular solution was used to fill the patch pipette to maximize RNA recovery. Cytoplasm was aspirated into the patch pipette, and expelled into a PCR tube which contained lysate buffer. The single cell RT-PCR reactions were performed using the “Single Cell-to-CT Kit for qRT-PCR” (Cat. No. 4458236, Thermo Fisher Scientific). The mRNA levels in individual neurons were quantified by StepOnePlus Real-Time PCR Systems (Thermo Fisher Scientific). PCR reactions were on triplicate cDNA samples. Predesigned gene expression assays were used for *Vglut2/solute carrier family 17* (Slc17a6-Mm00499876_m1) and *Gad67/glutamate decarboxylase 1* (Gad1 - Mm04207432_g1) genes and mouse 18S ribosomal RNA (Rn18s- Mm04277571_s1) genes. Data were evaluated with SDS 2.1 software. The comparative threshold cycle (CT) method was used to determine the relative amounts of transcripts.

### Quantification and Statistical Analysis

Prism6 and Origin were used for statistical analyses. No statistical methods were used to predetermine sample sizes, but our sample sizes are similar to those reported in previous studies. Data collection and processing were randomized or performed in a counter-balanced manner. Normality was tested by the Shapiro-Wilk test. Equal variances were assessed by F-test. Data are represented as the mean ± SEM, unless otherwise stated. For LORR, Fisher’s exact test or a two-tailed unpaired t test was performed. For the behavioral experiments, two-way ANOVA (time and treatment factors) was performed with t tests where appropriate. *P value*s are shown when they are less than 0.05. Mice were excluded from the analysis if the histology did not confirm significant AAV transgene expression in the LHb, or if the transgene expression spread beyond the target region. Investigators were not blinded to treatment.

## Supplemental Information

Document S1. Figures S1–S4

Document S2. Article plus Supplemental Information
