# Supplementary material for: Excitatory Pathways from the Lateral Habenula Enable Propofol-Induced Sedation
Source: Curr Biol. 2018 Feb 19;28(4):580–587.e5. doi: 10.1016/j.cub.2017.12.050 (PMC5835141; doi:10.1016/j.cub.2017.12.050)
Supplement: Document S1. Figures S1–S4 [file mmc1.pdf]

**Current Biology, Volume *28***

# Supplemental Information

# Excitatory Pathways from the Lateral Habenula Enable Propofol-Induced Sedation

**Cigdem Gelegen, Giulia Miracca, Mingzi Z. Ran, Edward C. Harding, Zhiwen Ye, Xiao Yu, Kyoko Tossell, Catriona M. Houston, Raquel Yustos, Edwin D. Hawkins, Alexei L. Vyssotski, Hailong L. Dong, William Wisden, and Nicholas P. Franks**

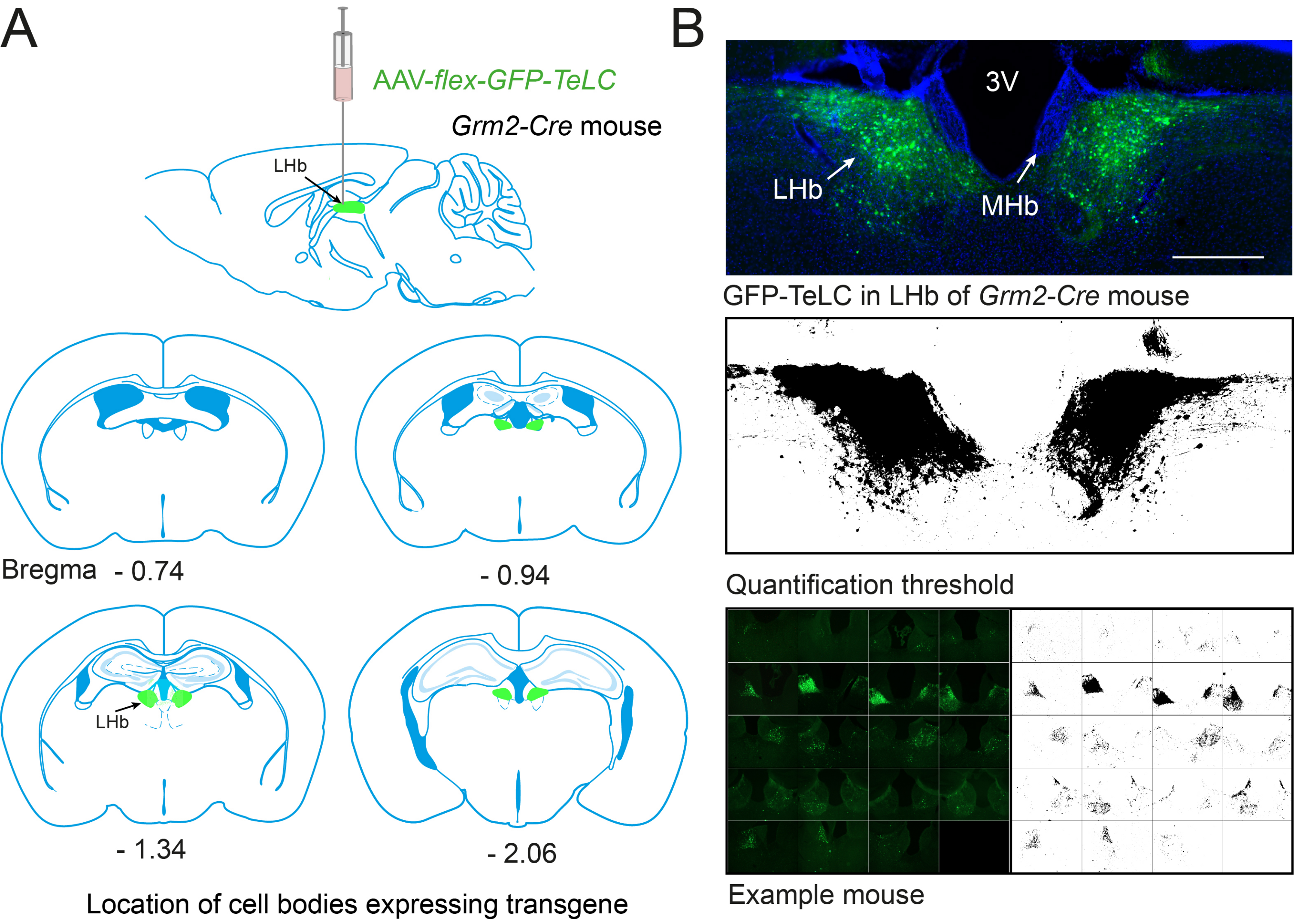
A
AAV-flex-GFP-TeLC
Grm2-Cre mouse
LHb
Bregma - 0.74
- 0.94
LHb
- 1.34
- 2.06
Location of cell bodies expressing transgene
B
3V
LHb
MHb
GFP-TeLC in LHb of Grm2-Cre mouse
Quantification threshold
Example mouse

**Figure S1. Related to Figure 1.** The extents of bilateral injections of AAV-*flex-GFP-TeLC* into the LHb of *Grm2-Cre* mice were determined by measuring integrated fluorescence intensity along the AP axis. (A) Example sections from Bregma -0.74 to -2.06 mm showing the location of *GFP-TeLC* expression (green). (B) Top: A typical coronal section showing GFP fluorescence in the LHb (note that this is the same image shown in **Figure 1A**); scale bar is 500 μm. Middle: All pixels above a chosen threshold are shown using the triangle algorithm and a custom macro in ImageJ (version1.50e; http://imagej.nih.gov/ij/). Bottom: The threshold was applied to a tiled montage of a single mouse brain.

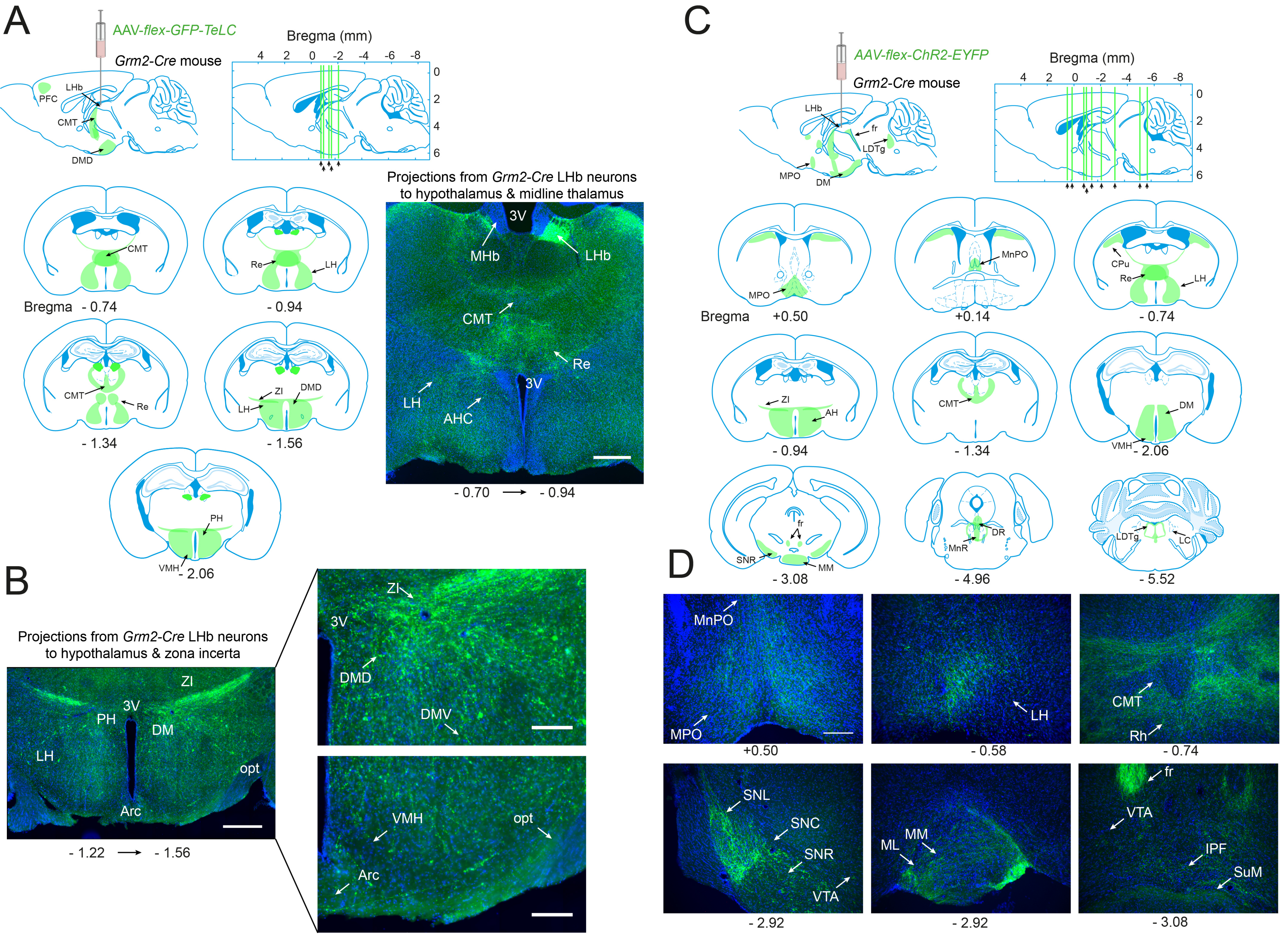
A
AAV-flex-GFP-TeLC
Grm2-Cre mouse
Bregma (mm)
PFC
LHb
CMT
DMD
Bregma - 0.74
- 0.94
- 1.34
- 1.56
- 2.06
Re
LH
ZI
PH
VMH
Projections from Grm2-Cre LHb neurons to hypothalamus & midline thalamus
3V
MHb
AHC
- 0.70 → - 0.94
B
Projections from Grm2-Cre LHb neurons to hypothalamus & zona incerta
DM
opt
Arc
- 1.22 → - 1.56
DMV
C
AAV-flex-ChR2-EYFP
fr
LDTg
MPO
MnPO
CPu
AH
SNR
MM
MnR
DR
LC
Bregma +0.50
+0.14
- 3.08
- 4.96
- 5.52
D
- 0.58
Rh
SNL
SNC
VTA
ML
IPF
SuM
- 2.92

**Figure S2. Related to Figure 1.** (A) Projections resulting from bilateral injections of AAV-*flex-GFP-TeLC* into the LHb of *Grm2-Cre* mice were mapped across the brain from Bregma -0.76 to -2.06 mm. The approximate distribution of projection targets is shown in the coronal schematics together with a picture of thalamic projections (scale bar 500 μm). (B) Hypothalamic projections (scale bar 500 μm) together with two expanded sections on the right (scale bars 200 μm). (C) Injections of AAV-*flex-ChR2-EYFP* were also used to map projections between Bregma +0.5 to -5.52 mm from the *Grm2-Cre* neurons in the LHb. Coronal schematics illustrate the approximate distribution of the projections. Where the fibre tract followed the same path as *GFP-TeLC* projections the same colour pattern was used for both figures. (B) Example images of distinct projection sites and local nuclei (scale bar 200 µm). AH, anterior hypothalamus; AHC, anterior hypothalamus central part; Arc, arcuate hypothalamic nucleus; CMT, central medial thalamus; CPu, caudate putamen; DM, dorsal medial hypothalamus; DMD, dorsal medial hypothalamus, dorsal part; DMV, dorsal medial hypothalamus, ventral part; DR, dorsal raphe nucleus; fr; fasciculus retroflexus; LC, locus coeruleus; LDTg, laterodorsal tegmental nucleus; LHb, lateral habenula; LH, lateral hypothalamus; MHb, medial habenula; MPO, medial preoptic nucleus; MnPO, median preoptic nucleus; MM, medial mammillary nucleus, medial part; ML, medial mammillary nucleus, lateral part; MnR, median raphe nucleus;opt, optic tract; PFC, pre-frontal cortex; PH, posterior hypothalamus; PO, preoptic area; Re, reuniens thalamic nucleus; SNR, substantia nigra, reticular part; SNC, substantia nigra, compact part; ZI, zona incerta; 3V, third ventricle; VMH, ventromedial hypothalamic nucleus.

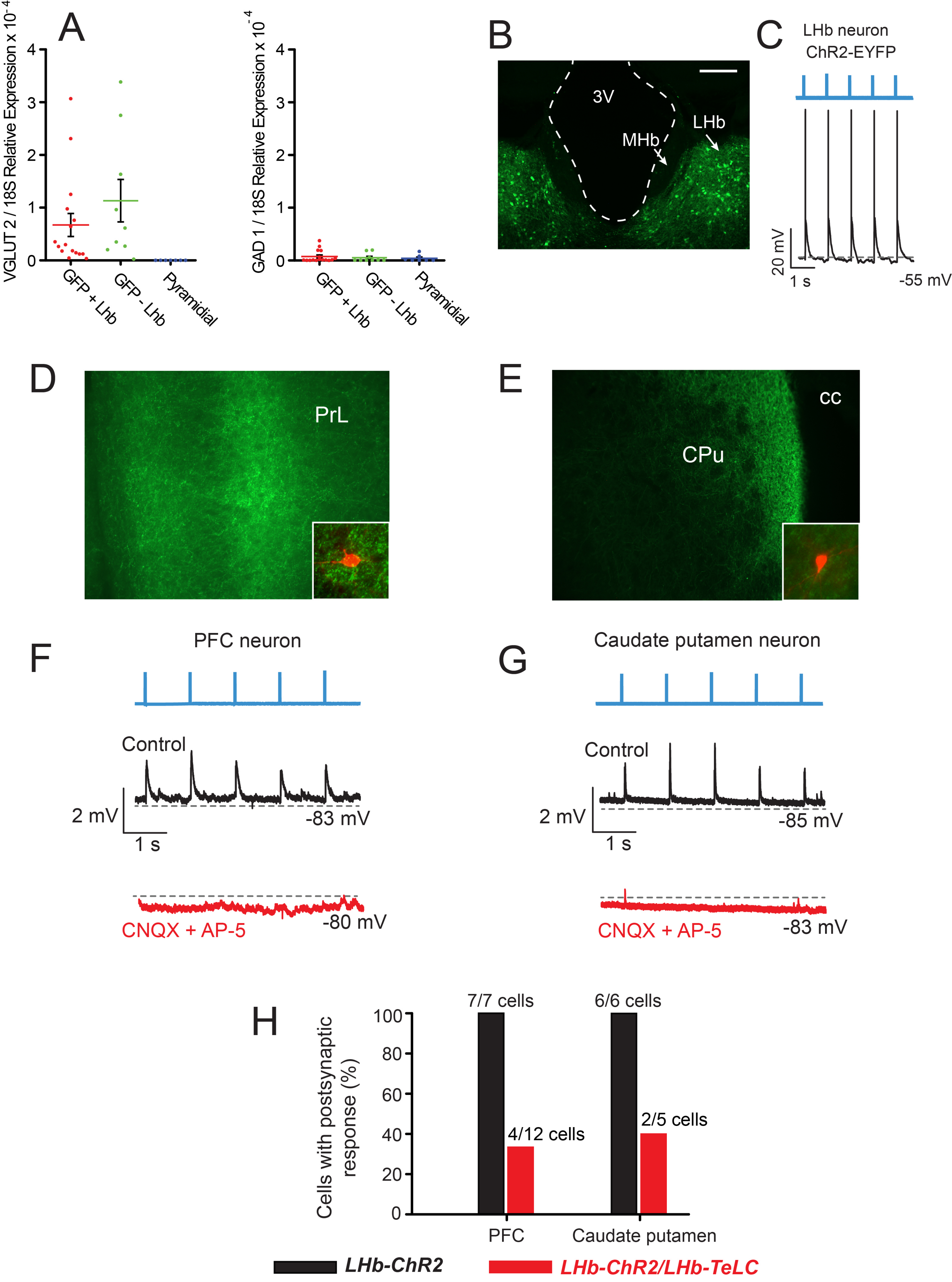
A
VGLUT 2 / 18S Relative Expression x 10$^{-4}$
GFP + Lhb
GFP - Lhb
Pyramidial
GAD 1 / 18S Relative Expression x 10$^{-4}$
B
3V
MHb
LHb
C
LHb neuron
ChR2-EYFP
20 mV
1 s
-55 mV
D
PrL
E
cc
CPu
F
PFC neuron
Control
2 mV
1 s
-83 mV
CNQX + AP-5
-80 mV
G
Caudate putamen neuron
Control
2 mV
1 s
-85 mV
CNQX + AP-5
-83 mV
H
Cells with postsynaptic response (%)
7/7 cells
6/6 cells
4/12 cells
2/5 cells
PFC
Caudate putamen
LHb-ChR2
LHb-ChR2/LHb-TeLC

**Figure S3. Related to Figure 1.** The output of *Grm2-Cre* LHb neurons is glutamatergic, and is blocked by TeLC. (A) Single-cell-patch RT-PCR analysis of transmitter phenotype of neurons (*n* = 26) in the LHb in acute brain slices from *LHb-ChR* mice. Both *Grm2-Cre* GFP-positive (*n* = 17) and GFP-negative (*n* = 17) neurons predominantly express the *Vglut2* gene, but with low levels of *Gad1* transcripts as well. As a control for specificity of the PCR, neocortical pyramidal neurons (*n* = 7) patched from the same slices, and run in parallel assays, do not contain *Vglut2* or *Gad1* transcripts. The horizontal lines represent the means and the error bars represent the SEMs. (B) Bilateral injection of AAV-*flex-ChR2-EYFP* into the LHb region of *Grm2-Cre* mice; scale bar is 200 μm. (C) An example whole-cell current-clamp recording from a LHb neuron expressing ChR2-EYFP. Brief (2-ms) light pulses (blue lines) consistently produced action potentials (*n* = 18 neurons, 3 mice). The *Grm2-Cre* LHb axons project to the infralimbic (PrL) prefrontal cortex (D) and dorsal caudate-putamen (CPu). Cc, corpus callosum. (E) The insets show examples of biocytin-filled neurons post recording. Light stimulation of ChR2-EYFP positive LHb axon terminals evoked EPSPs (black traces) in postsynaptic prefrontal cortex neurons (F) and dorsal-lateral caudate-putamen neurons (G), which were blocked by CNQX and AP-5 (red traces). (H) Percentage of cells with detected post-synaptic EPSPs in *LHb-ChR2* mice (black) and *LHb-ChR2/LHb-TeLC* mice (red) in prefrontal cortex and caudate-putamen respectively.

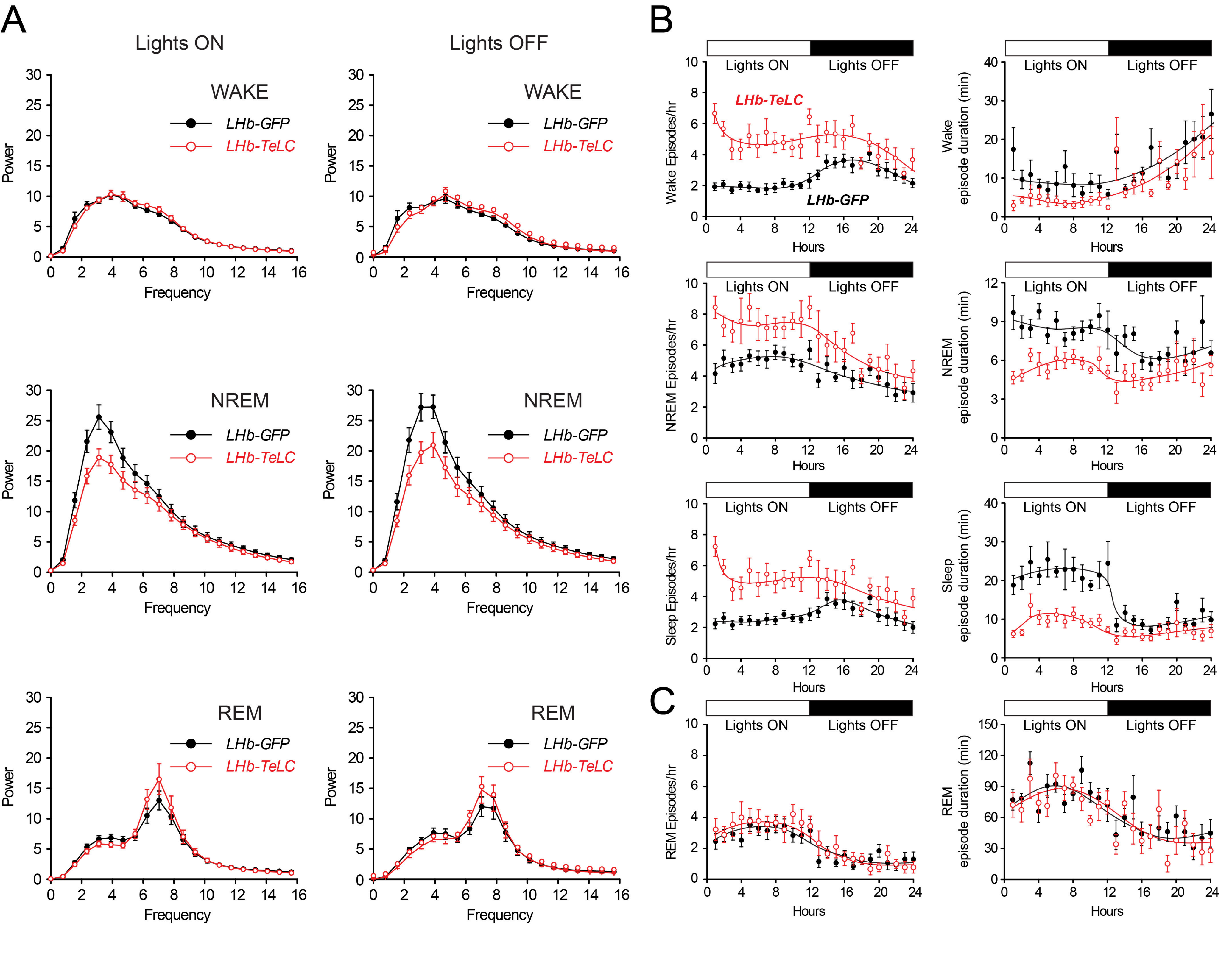

A
Lights ON
Lights OFF
WAKE
NREM
REM
LHb-GFP
LHb-TeLC
Power
Frequency
B
C
Lights ON
Lights OFF
LHb-TeLC
LHb-GFP
Wake Episodes/hr
Wake episode duration (min)
NREM Episodes/hr
NREM episode duration (min)
Sleep Episodes/hr
Sleep episode duration (min)
REM Episodes/hr
REM episode duration (min)
Hours

**Figure S4. Related to Figure 4.** Sleep in *LHb-TeLC* mice compared with *LHb-GFP* mice. (A) Power spectra of vigilance states for the *LHb-GFP* and *LHb-TeLC* mice. The Fourier transform power spectra for the *LHb-GFP* and *LHb-TeLC* mice were no different during Wake and REM states, but did show slightly lower power over delta frequencies (1-4Hz) during NREM. This was true during both “Lights ON” ($P$ = 0.022, $t$ = 2.45, $df$ = 21, $n$ = 13 *LHb-GFP* mice and 10 *LHb-TeLC* mice; unpaired two-tailed $t$-test) and “Lights OFF” ($P$ = 0.020, $t$ = 2.51, $df$ = 21, $n$ = 13 *LHb-GFP* mice and 10 *LHb-TeLC* mice; unpaired two-tailed $t$-test). (B) For Wake, NREM and Sleep (defined as consolidated bouts of NREM and REM) the number of episodes per hour (left-hand panels) are shown over a 24-hour period while the duration of episodes is shown on the right hand panels. (C) The number of REM episodes per hour (left) and durations of episodes over a 24-hour period ($n$ = 13 *LHb-GFP* mice and $n$ = 9 *LHb-TeLC* mice). Symbols in panels A, B and C represent means ± SEMs.
